# Design of Acrylate-Terminated Polyurethane for Nylon Seamless Bonding Fabric Part I: Design of the End-Capping Thermoplastic Polyurethane Adhesive with Acrylate Copolymer

**DOI:** 10.3390/polym14194079

**Published:** 2022-09-28

**Authors:** Jiong-Bo Chen, Sheng-Yu Lin, Naveed Ahmad, Chung-Feng Jeffrey Kuo

**Affiliations:** Department of Materials Science and Engineering, National Taiwan University of Science and Technology, Taipei 10607, China

**Keywords:** thermoplastic polyurethane, mass polymerization, N,N-dimethylacrylamide, end capping

## Abstract

This series of studies aims to design acrylate-terminated polyurethanes for use in nylon seamless bonded fabrics. The first part used N,N-dimethylacrylamide (DMAA) and methyl methacrylate (MMA) to replace the chain extender in polyurethane synthesis as end-capping agent to synthesize thermoplastic polyurethane (TPU) adhesive. The molecular weight of the TPU is controlled to further influence the mechanical and processing properties of the polyurethane. Here, polytetramethylene ether glycol (PTMG) and 4,4-methylene diphenyl diisocyanate (MDI) were polymerized, and then a blocking agent was added thereto. The results show that the characteristic peaks of benzene ring and carbamate of TPU adhesive are at 1596 cm^−1^ and 1413 cm^−1^, respectively, while the characteristic peaks of DMAA are at 1644 cm^−1^ and 1642 cm^−1^ in the FT-IR spectrum. There is an absorption peak –N=C=O– which is not shown near 2268 cm^−1^, which proves that the structure of TPU contains the molecular structure of capping agent, PTMG and MDI. When the DMAA concentration in the capping agent was increased from 3.0 wt% to 10 wt%, the –C=O (H-bond) area percentage of hydrogen bonds formed at 1711 cm^−1^ increased from 41.7% to 57.6%, while the –NH (H bond) produced at 3330 cm^−1^ increased from 70% to 81%. These phenomena suggest that increasing the concentration of DMAA capping agent can effectively promote the formation of complex supramolecular network structures by hydrogen bonding in TPU. The content and concentration of the capping agent affects the molecular weight of the TPU. Chain growth is terminated when molecular weight growth can be effectively controlled and reduced. It was observed in thermal analysis that with increasing DMAA concentration in the molecular structure, the concentration of capping agent in TPU, hydrogen bonding force between hard segments, melting point (Tmh) and melting enthalpy (ΔH) all increased the capping agent. The pyrolysis temperature of TPU is increased by 10–20 °C.

## 1. Introduction

Thermoplastic polyurethanes (TPUs) are (AB)n linear block polymers. Molecular segments with better softness endow TPU with ductility and flexibility, so they are called soft segments, while hard segments provide rigidity to TPU [1]. TPU can synthesize TPU products with different physical and mechanical properties by adjusting the ratio of soft segment to hard segment [2]. Compared with the soft segments in the molecular structure, the hard segments show low polarity and interfacial quality. Therefore, TPU has a microphase-separated state. Compared with different plastics and rubbers, TPU has excellent physical and chemical properties, high wear resistance, high flexibility, high tensile strength, high ductility and excellent elasticity [3]. The main raw materials are polyols, isocyanates and chain extenders. Through the selection and ratio adjustment of raw materials, TPU with desired properties is prepared. In order to speed up the reaction, increase some properties, and reduce the cost of the product, corresponding excipients are sometimes required. The molecular weight (Mn) of the low degree of polymerization polyol used for the polyurethane is 1000 g/mol to 3000 g/mol. The type, polarity, crystallinity and molecular weight of the polyol with low polymerization degree have a significant effect on the resilience, tensile and low temperature properties of TPU. The common types of isocyanates are TDI and MDI; TDI exists as two isomers: 2,4- and 2,6-TDI and is used in the manufacture of flexible PU foams, coatings, sealants and elastomers. TDI is low in price and has high reactivity, but its irritant and volatile toxicity are also relatively high. The MDI used here can be divided into 4,4-MDI and 2,4-MDI. The molecular structure of 4,4-MDI is symmetric, while the molecular structure of 2,4-MDI is asymmetric. Therefore, 2,4-MDI can reduce the crystallinity of the hard segment and enhance the compatibility between the soft segment and the hard segment. The chemical structure of isocyanate affects the reactivity, and the main factors affecting the reactivity are the electronic effect and the steric hindrance effect. The conjugation of benzene rings makes aromatic isocyanates more reactive than aliphatic isocyanates [4,5]. Two isocyanates of isocyanate molecular structure have relative reactivity in different chemical environments [6,7,8]. Eceiza et al. [9] demonstrated that the structure and molecular weight of low-degree-of-polymerization polyols influence the reaction rate through autocatalysis. When the main structure of low-polymerization polyols is the same but the molecular weight is different, the corresponding reaction rate constant decreases with the increase of molecular weight [9,10]. Kulkarni et al. [11] synthesized TPU using polyisobutylene and PTMG, and analyzed thermal and mechanical properties. The thermal properties show that the hard segments of the TPU are completely amorphous, and the hard and soft segments are clearly mixed. The results show that after adjusting the ratio of hard segment to soft segment, the mechanical properties of TPU are comparable to conventional polyether TPU. Schneider et al. [12] demonstrated that an important reason for the high strength of TPU is the phase-separated structure, but if the degree of phase separation is too high, the tensile strength of the material will decrease. Middle and so on [13] used polypropylene glycol (PPG), MDI and 1,4-butanediol (BDO) to synthesize TPU, and microphase separation occurred when the hard segment content was 50 wt%. The Young’s modulus and tensile strength of TPU increased dramatically, and the surface free energy of TPU increased due to microphase separation.

The purpose of this study is to investigate the influence of the molecular structure and hydrogen bonding of the end-capping agent, and to analyze the enhanced degree of hydrogen bonding of the TPU molecular segments, thereby improving the tensile strength, peel strength and shear strength. This modified TPU is used in nylon to reinforce the fibers.

The end-capping agent was synthesized using methyl methacrylate (MMA) and N,N-dimethylacrylamide (DMAA) as the main raw materials. Azobisisobutyronitrile (AIBN) and 2-mercaptoethanol (ME) were used as initiator and chain transfer agent, respectively. The capping agent was synthesized by a radical chain transfer reaction mechanism. Using polybutylene glycol, 4,4′-diphenylmethane diisocyanate and end-capping agent as raw materials, TPU is synthesized by bulk polymerization process. The content of the capping agent is adjusted to control the molecular weight of the TPU, and the DMAA concentration of the capping agent is adjusted to control the mechanical properties of the TPU. After TPU is compounded with nylon fabric, different characterization techniques such as FT-IR, GPC, TGA, DSC, DMA, etc., are used to analyze the functional group, hydrogen bond state, molecular weight state, pyrolysis state, melting point, melting enthalpy and glass transition of TPU temperature.

## 2. Experimental Method and Materials

### 2.1. Materials

Methyl methacrylate (MMA), molecular weight: 110.12 g/mole, from: Sigma-Aldrich (St. Louis, MO, USA). N,N-dimethylacrylamide (DMAA), molecular weight: 99.13 g/mole, from: Sigma-Aldrich. Azobisisobutyronitrile (AIBN), molecular weight: 164.21 g/mole, from: Sigma-Aldrich. 2-Mercaptoethanol (ME), molecular weight: 262 g/mole, from: Sigma-Aldrich. Ethyl acetate (EAc), molecular weight: 118 g/mole, from: Sigma-Aldrich. 4,4′-diphenylmethane diisocyanate (MDI), molecular weight: 250 g/mole, from: Sigma-Aldrich. Polytetramethylene glycol (PTMG), molecular weight: 1000 g/mole, from: Sigma-Aldrich.

### 2.2. Experimental Method

**Figure 1:**
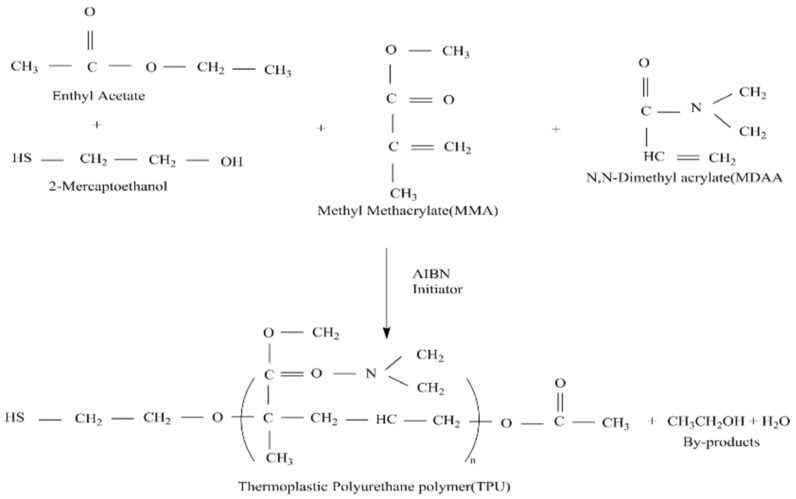
Schematic diagram of self-made capping agent design.

A solution was prepared by dissolving methyl methacrylate (MMA) in ethyl acetate (EAC). In this solution, N,N-dimethylacrylate (DMMA) and 2-mercaptoethanol (ME) were added as polymerization terminators, and the reaction temperature was fixed at 70 °C. The azobisisobutyronitrile (AIBN) was used as a catalyst to complete the reaction. Eventually, the capping agent based on methyl methacrylate (MMA) and N,N-dimethacrylate (DMAA) was synthesized after 24 h of polymerization. The structure of the capping agent is shown in Figure 1. The formula for synthesis of end-capping agent is shown in Table 1.

The MMA obtained by polymerization is 37 wt%, 35 wt%, 33 wt% and 30 wt%, respectively.

The PTMG and MDI were put in the reaction bulb in turn and heated to 80 °C in nitrogen environment, and polymerized by the mechanical agitator at speed of 200 rpm. After 1 h of polymerization, the end-capping agent was put in the reaction bulb. The TPU prepolymer was formed after 1 h of reaction (total reaction time was 2 h). The TPU prepolymer was ripened at room temperature for 24 h, and put in the oven at 80 °C to remove the solvent. The generated data are shown in Table 2.

**Figure 2:**
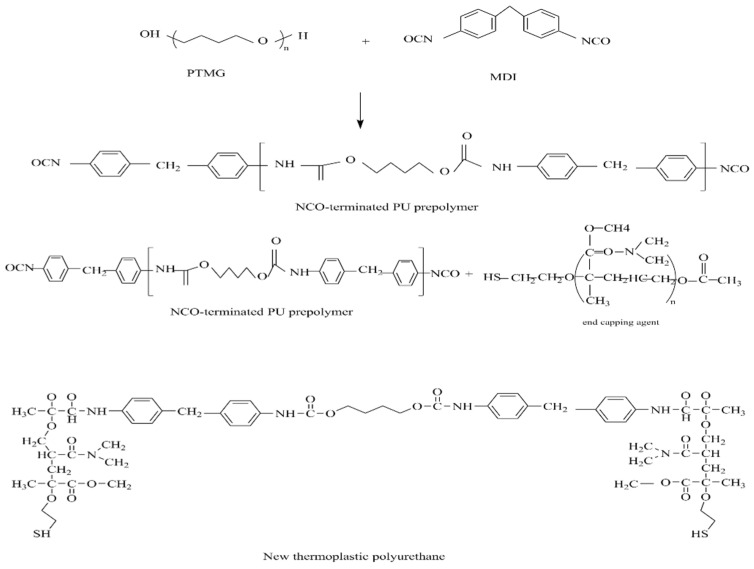
New thermoplastic polyurethane.

The compounding end-capping agents D (03, 05, 07, 10) were added to the polyurethane prepolymer, respectively. The addition amount was shown in Table 2. The reaction temperature was below 80 °C and continued to stir for 2 h. After the end-capping, a new type of thermoplastic polyurethane can be obtained and the structure is shown in Figure 2.

Afterwards, the TPU was cut into a 1.0 cm × 15 cm strip (thickness: 150 μm), and the nylon fabric was cut to the size of 2.54 cm × 18 cm. They were bonded according to the following processing conditions, and the bonding conditions included:

(a) Bonding temperature: 150 °C.
(b) Bonding pressure: 1.0 kg/cm^2^.
(c) Bonding time: 20 s.
(d) Standing time after bonding: 20 min for cooling and stabilization.

### 2.3. Instrumental Analysis

#### 2.3.1. Test for Functional Group and H-Bond State of TPU

The functional group and H-bond state of TPU were measured by using the Fourier transform infrared spectroscopy (FT-IR, Model: Digilab FTS-1000, Hopkinton, MA, USA). The range of wave number scanned by ATR was 4000 cm^−1^ to 650 cm^−1^, and the resolution was 2 cm^−1^. The average of 16 scans was taken.

#### 2.3.2. Test for Molecular Weight and Molecular Weight Distribution of TPU

The molecular weight and molecular weight distribution of TPU were measured by using the gel permeation chromatography (GPC, Model 500, Theale, UK), including a reflective index (RI) detector (Schambeck RI2000, DURATEC ANALYSENTECHNIK GmbH, Hockenheim, Germany) and two tubular columns in a series of Jordi gel DVB mixed beds and measuring the molecular weight distribution in relation to PS standard on 30 °C 10,000 Å bed. The carrier solvent was tetrahydrofuran, and the flow velocity was 1.0 mL/min.

#### 2.3.3. Test for Pyrolysis Temperature of TPU

The pyrolysis temperature of TPU was measured by using the thermogravimetric analysis (TGA, model: TA Instruments-DuPont Q500, Wilmington, DE, USA). The amount of sample was 5.0 mg to 8.0 mg, in N2, the temperature increased from 50 °C to 700 °C, the heating rate was 10 °C/min, and the TGA was performed.

#### 2.3.4. Test for Melting Point and Melting Enthalpy of TPU

The melting point and melting enthalpy of TPU were measured by using the differential scanning calorimeter (DSC, model: Perkin Elmer DSC 4000, Waltham, MA, USA). The test specimen was 5.0 mg–8.0 mg and sealed in an Al tray. The test conditions are described below, (a) heating condition: from −40 °C to 240 °C, the heating rate is 10 °C/min; and (b) cooling condition: from 240 °C to −40 °C, the cooling rate is 10 °C/min.

#### 2.3.5. Test for Glass Transition Temperature and Storage Modulus of TPU

The glass transition temperature and storage modulus of TPU were measured by using the dynamic mechanical analyzer (DMA, TA Instruments Q800, New Castle, DE, USA). In stretching mode, the frequency was 1 Hz, the temperature rose from −80 °C to 50 °C, the heating rate was 2.0 °C/min. The sample size was 20 × 5 × 0.2 mm (L × W × H).

## 3. Results and Discussion

### 3.1. Analysis of Functional Groups of TPU

**Figure 3:**
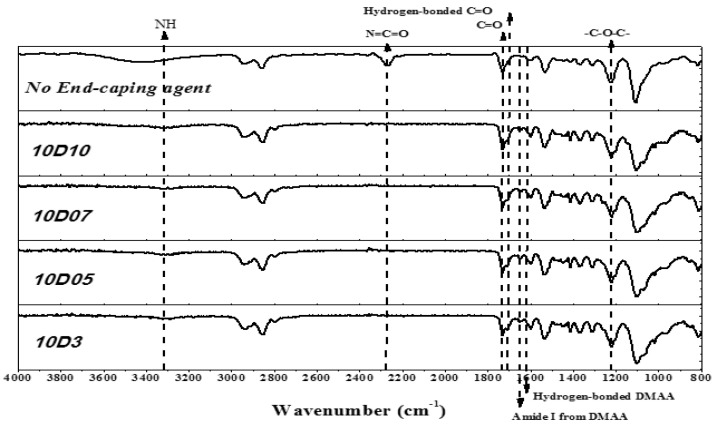
FT-IR spectra of TPU.

**Figure 4:**
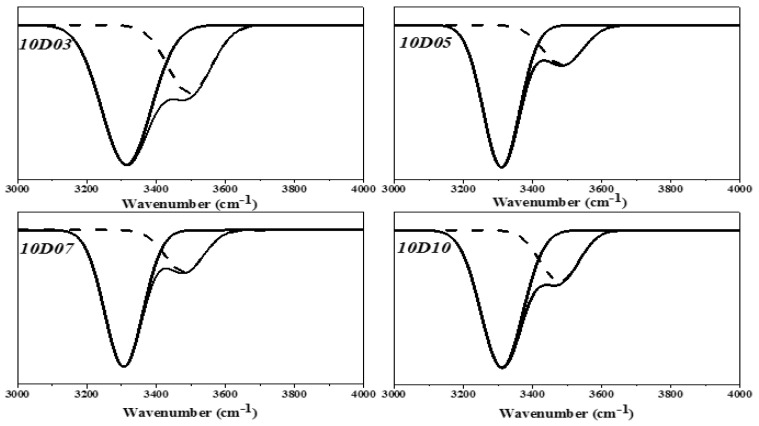
Simulated peak splitting diagrams of FT-IR spectra of –C=O group.

**Figure 5:**
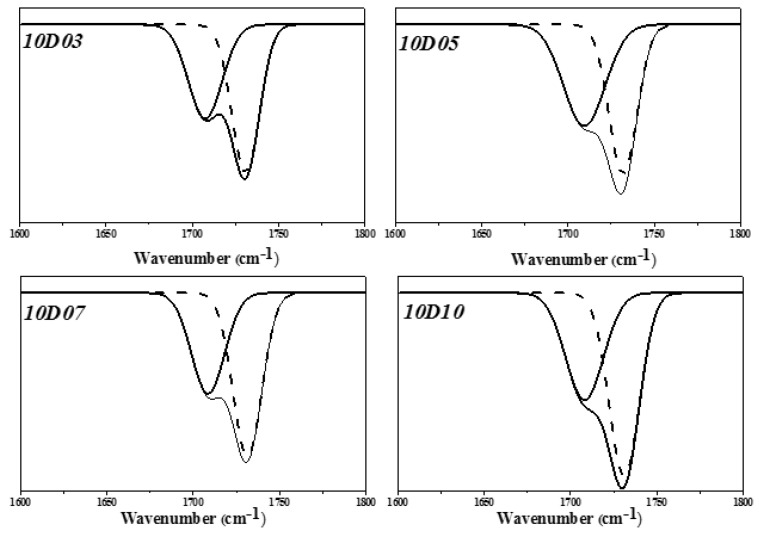
Simulated peak splitting diagrams of FT-IR spectra of –NH group.

This study used PTMG and MDI as the base of synthesizing TPU, and used D03, D05, D07 and D10 as end-capping agents, respectively, to synthesize TPU. The functional groups of TPU were analyzed by FT-IR. As shown in Figure 3, the benzene ring shows the stretching vibration absorption peak at 1596 cm^−1^ and 1413 cm^−1^. The –C–H showed the bending vibration absorption peak at 1529 cm^−1^. The –C–N showed the stretching vibration absorption peak at 1308 cm^−1^. The –C–O showed the stretching vibration absorption peak at 1218 cm^−1^ [14]. Additionally, the main characteristic peak Amide I of DMAA is observed at 1644 cm^−1^ and 1642 cm^−1^ [15,16]. The H-bond generated by –C=O group of DMAA is observed at 1613 cm^−1^ [17]. The absorption peak of –C=O is observed at 1730 cm^−1^ and split into double peak, meaning the H bonding strength of –NH is very high. It is observed in the FT-IR spectra that the –N=C=O– stretching vibration absorption peak near 2268 cm^−1^ of the TPU sample with end-capping agent completely disappears, but the –NH absorption peak and Amide I absorption peak of DMAA occurred. This suggested that the TPU structure contained the molecular structures of end-capping agent, PTMG and MDI. In the FT-IR spectra, the absorption peak regions of –C=O group and –NH group are 1650 cm^−1^–1780 cm^−1^ (–C=O) and 3200 cm^−1^–3500 cm^−1^ (–NH), respectively, which are used as the feature regions for observing the hydrogen bonding. During hydrogen bonding, the two groups are disintegrated into two absorption peaks and move towards low wave number. The FT-IR spectra in Figure 4 and Figure 5 showed the absorption peaks of –C=O group and –NH group, respectively. Origin 8.0 software was used to simulate splitting peak. Figure 4 showed the simulated peak splitting result of –C=O group absorption peak, and the –C=O groups of free (–C=O (free)) and H-bond (–C=O (H-bond)) are at 1732 cm^−1^ and 1711 cm^−1^. Figure 5 showed the simulated peak splitting result of –NH group absorption peak, and the –NH groups of free (–NH (free)) and H-bond (–NH(H-bond)) are at 3400 cm^−1^ to 3200 cm^−1^ [17,18]. As shown in Table 3, the increase in the DMAA concentration of end-capping agent has a significant effect on the H-bond area percent generated by –C=O and –NH. The results are described below: (a) when the DMAA concentration of end-capping agent increased from 3.0 wt% to 10 wt%, the –C=O (H-bond) of H-bond generated at 1711 cm^−1^ increases from 41.7% to 57.6%; the free –C=O (free) at 1732 cm^−1^ decreased from 58.3% to 42.4%; (b) when the DMAA concentration of end-capping agent increased from 3.0 wt% to 10 wt%, the –NH (H-bond) of H-bond generated at 3330 cm^−1^ increased from 70% to 81%; the free –NH (free) at 3440 cm^−1^ decreased from 30% to 19%. As the DMAA concentration in the end-capping agent increased, the ability of –C=O group to receive protons and form H-bond is enhanced. The formation of H-bond is promoted, and a complex supermolecular network structure is formed in the TPU. However, the –C=O group in the molecular structure of DMAA has relatively strong ability to attract protons. The –NH of carbamate has relatively strong ability to give protons, so that more H-bonds are generated. It means that the stronger the ability of the proton donor to give protons, the higher is the electronegativity of proton acceptor, and the H-bond is likely to be formed due to difference of polarity, which ultimately increased the bond energy [18,19].

### 3.2. Analysis of Molecular Weight and Distribution Coefficient of TPU

**Figure 6:**
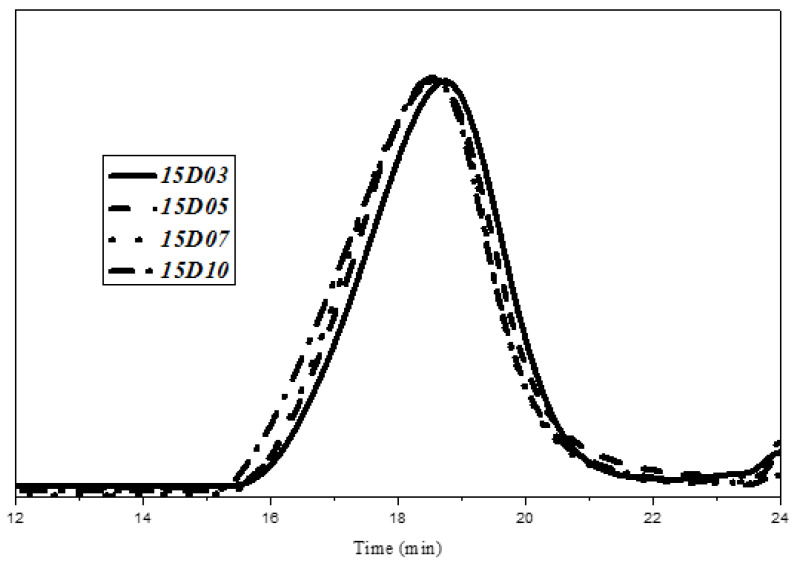
GPC curve of TPU with end-capping agent at concentration of 5.0 phm.

**Figure 7:**
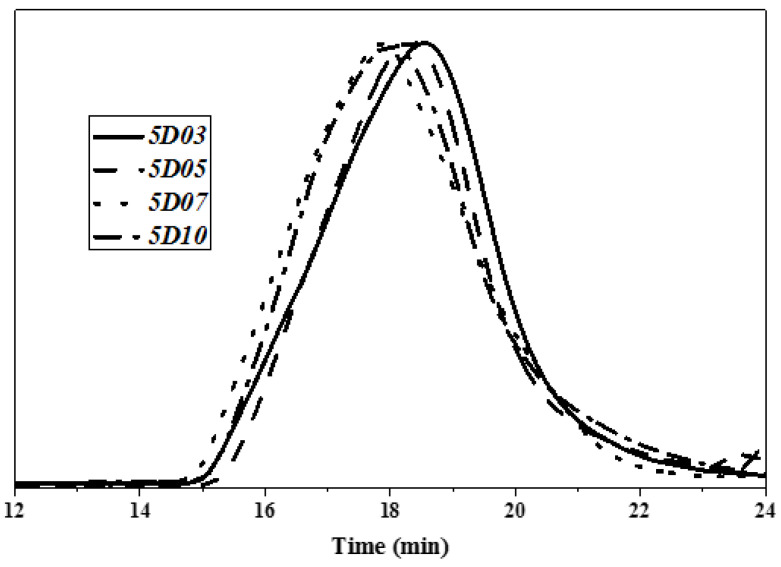
GPC curve of TPU with end-capping agent at concentration of 10 phm.

**Figure 8:**
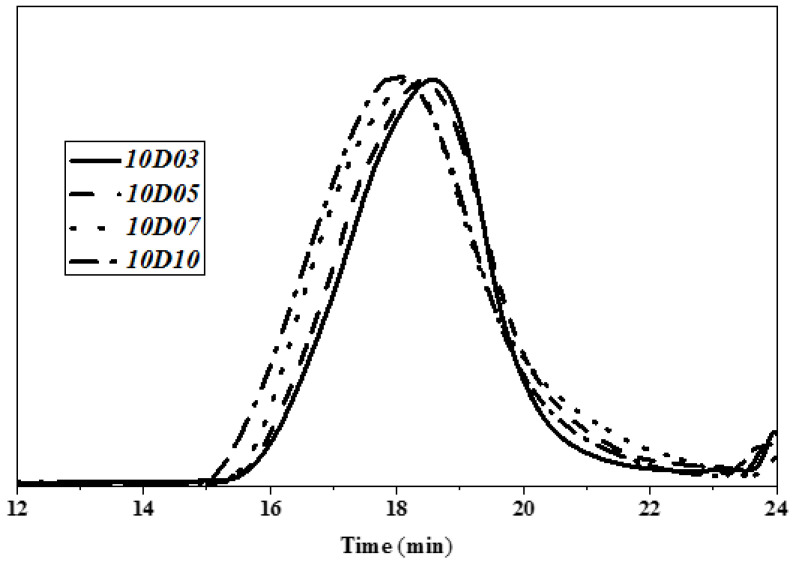
GPC curve of TPU with end-capping agent at concentration of 15 phm.

**Figure 9:**
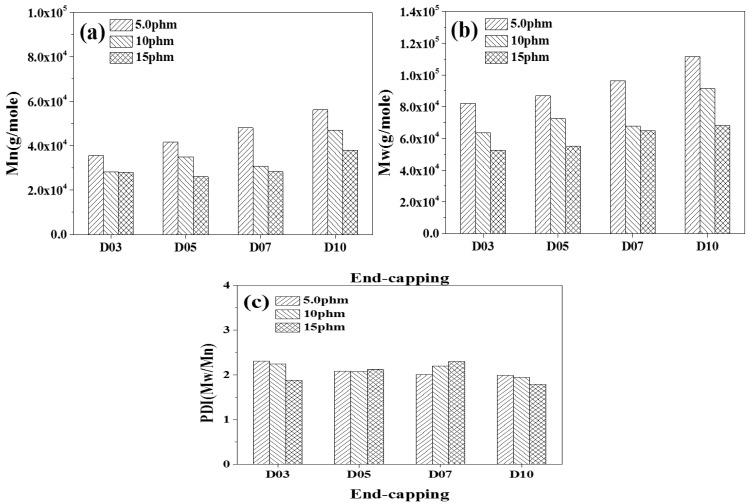
Tendency chart of molecular weight of TPU, (**a**) number average molecular weight (Mn), (**b**) weight average molecular weight (Mw), (**c**) molecular weight distribution coefficient (PDI (Mw/Mn)) of TPU.

The relative molecular weight and molecular weight distribution of TPU have important effects on mechanical properties and post-processing properties. The tensile strength of TPU increases with increasing molecular weight of TPU. When the molecular weight exceeds a certain molecular weight range, the processing temperature of TPU increases, which affects the later processability of TPU. In this study, TPU was synthesized based on PTMG and MDI, and TPU was synthesized with D03, D05, D07 and D10 as capping agents, respectively. The weight average molecular weight (Mw), number average molecular weight (Mn) and molecular weight distribution coefficient (PDI) of TPU were analyzed by GPC. Figure 6, Figure 7 and Figure 8 are the GPC curves of TPU synthesized with three different contents of end-capping agents, and Figure 9 is the molecular weight trend diagram of the GPC curve. As shown in Figure 9 ((a) number average molecular weight (Mn) (b) weight average molecular weight (Mw), (c) molecular weight distribution coefficient (PDI (Mw/Mn)) of TPU) and Table 4, the Mw and Mn of TPU varied with the concentration of DMAA in the capping agent. It was observed that when the capping agent content was 5.0 phm, DMAA increased from 3.0 wt% to 10 wt%, Mw increased from 82,000 g/mole to 111,580 g/mole, Mn increased from 35,520 g/mole to 56,100 g/mole; when increasing to 10 phm, DMAA concentration increased from 3.0 wt% to 10 wt%, Mw increased from 63,455 g/mole to 91,540 g/mole, Mn increased from 28,270 g/mole to 47,020 g/mole; and when the capping agent content increased to 15 phm, the DMAA concentration increased from 3.0 wt% to 10 wt%, the Mw increased from 52,480 g/mole to 68,080 g/mole, and the Mn increased from 27,960 g/mole to 37,940 g/mole. The molecular weight of the TPU increased with increasing DMAA concentration in the capping agent structure. The molecular weight of TPU is affected by the steric hindrance effect produced by –N(CH_3_)_2_ of DMAA, which inhibits the formation of covalent bonds between –OH of the capping agent and isocyanate [20,21]. The molecular weight distribution (PDI) of TPU indirectly proves that the steric hindrance effect produced by N(CH3)_2_ leads to the uneven molecular weight distribution. In addition, the Mw and Mn of TPU varied with the content of capping agent, as described below: (a) TPU synthesized with capping agent D03, the content of capping agent was increased from 5.0 phm to 15 phm, the Mw of TPU decreased from 82,000 g/mole to 52,480 g/mole, and the Mn decreased from 35,520 g/mol to 27,960 g/mole Moore; (b) TPU synthesized with capping agent D05, the content of capping agent was increased from 5.0 phm to 15 phm, the Mw of TPU decreased from 86,810 g/mole to 55,080 g/mole, and the Mn decreased from 41,530 g/mol to 26,020 g/mole Moore; (c) TPU synthesized with capping agent D07, the content of capping agent was increased from 5.0 phm to 15 phm, the Mw of TPU was decreased from 96,330 g/mole to 64,730 g/mole, and the Mn was decreased from 47,980 g/mole to 28,300 g/mole; (d) synthesis of TPU with end-capping agent D10 series, the content of end-capping agent was increased from 5.0 phm to 15 phm, the Mw of TPU decreased from 111,580 g/mole to 68,080 g/mole, and the Mn decreased from 56,100 g/mol to 37,940 g/mol. The results show that the –OH of the blocking agent and the isocyanate group form a covalent bond, thus achieving the purpose of blocking.

### 3.3. Analysis of Pyrolysis Temperature of TPU

**Figure 10:**
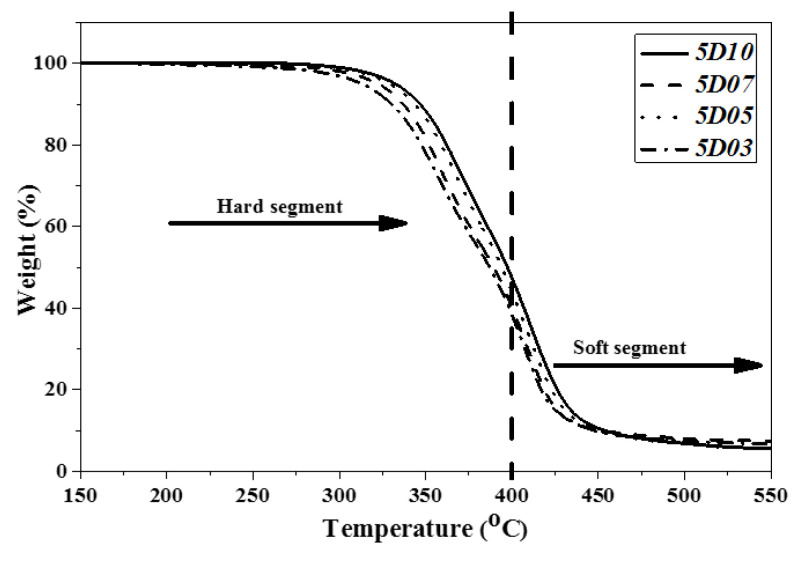
TGA curve of TPU with end-capping agent at concentration of 5.0 phm.

**Figure 11:**
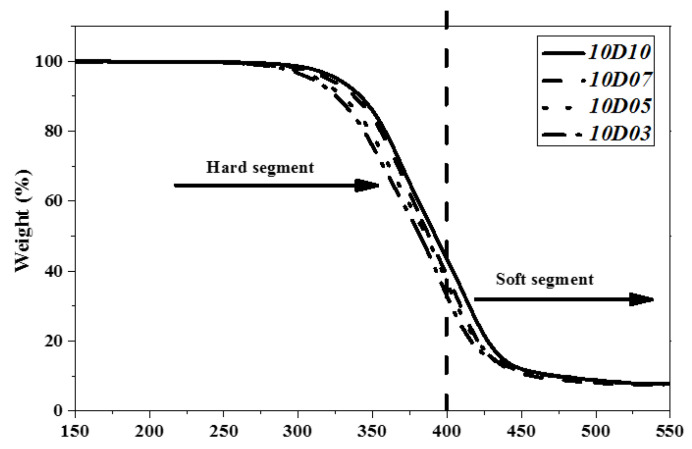
TGA curve of TPU with end-capping agent at concentration of 10 phm.

**Figure 12:**
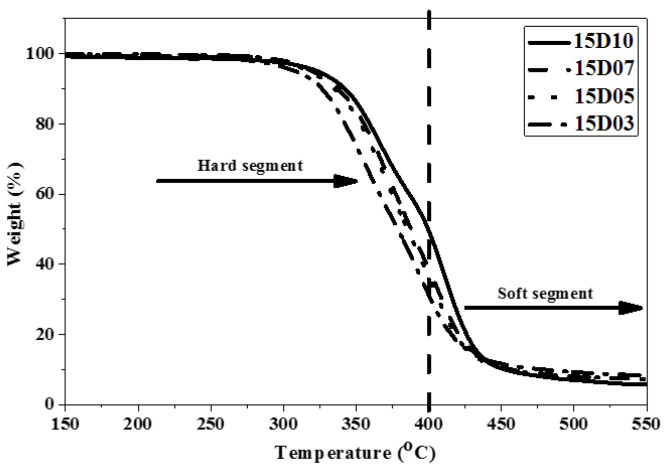
TGA curve of TPU with end-capping agent at concentration of 15 phm.

This study used PTMG and MDI, which are the bases for the synthesis of TPU, and used D03, D05, D07 and D10 as end-capping agents, respectively, to synthesize TPU. The temperature of pyrolyzing 5.0 wt% (Td5) and the temperature of pyrolyzing 10 wt% (Td10) and maximum decomposition rate of TPU were analyzed by using TGA as described in Table 5. Figure 10, Figure 11 and Figure 12 show the TGA curves of synthetic TPU with three contents of end-capping agent, respectively. The DMAA concentration in the end-capping agent has a significant effect on the TPU decomposition temperature, described below: (a) when the content of end-capping agent is 5.0 phm, the DMAA concentration in the end-capping agent increases from 3.0 wt% to 10 wt%, and the pyrolysis temperature rises from 312 °C to 332 °C; (b) when the content of end-capping agent is 15 phm, the DMAA concentration in the end-capping agent increases from 3.0 wt% to 10 wt%, and the pyrolysis temperature rises from 309 °C to 323 °C. In previous studies [22,23], the –N(CH_3_)_2_ grafted copolymer is decomposed at 300–550 °C. When the DMAA is admitted in the polymer, the pyrolysis temperature of the polymer rises. Because the –C=O group and polar group, such as –OH group, of DMAA form strong H-bond, the thermal stability of the polymer is improved [24,25]. In TGA curve, the pyrolysis of TPU can be divided into the pyrolysis of hard segment at 200 °C to 400 °C and the pyrolysis of soft segment at 400 °C to 500 °C [26,27]. Additionally, the pyrolysis temperature is related to molecular weight of the polymer [28], described below: (a) TPU synthesized with end-capping agent D03, the content of end-capping agent increases from 5.0 phm to 15 phm, and the pyrolysis temperature of TPU drops from 312 °C to 309 °C; (b) TPU synthesized with end-capping agent D05, the content of end-capping agent increases from 5.0 phm to 15 phm, and the pyrolysis temperature of TPU drops from 328 °C to 317 °C; (c) TPU synthesized with end-capping agent D07, the content of end-capping agent increases from 5.0 phm to 15 phm, and the pyrolysis temperature of TPU drops from 322 °C to 320 °C; (d) TPU synthesized with end-capping agent D10, the content of end-capping agent increases from 5.0 phm to 15 phm, and the pyrolysis temperature of TPU drops from 332 °C to 323 °C.

### 3.4. Analysis of Melting Point and Melting Enthalpy of TPU

**Figure 13:**
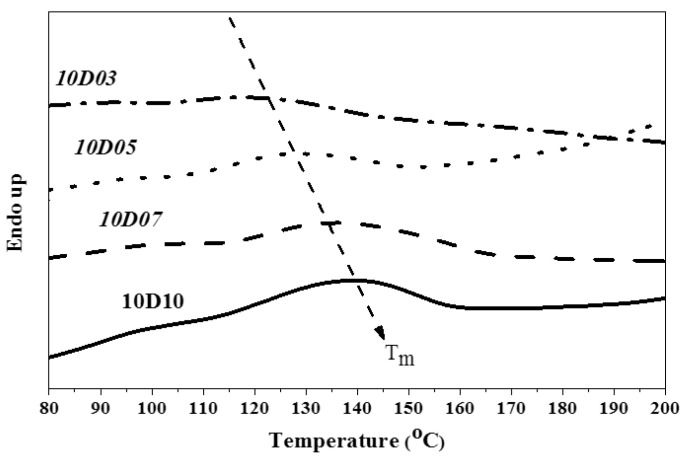
DSC curve of TPU with end-capping agent at concentration of 5.0 phm.

**Figure 14:**
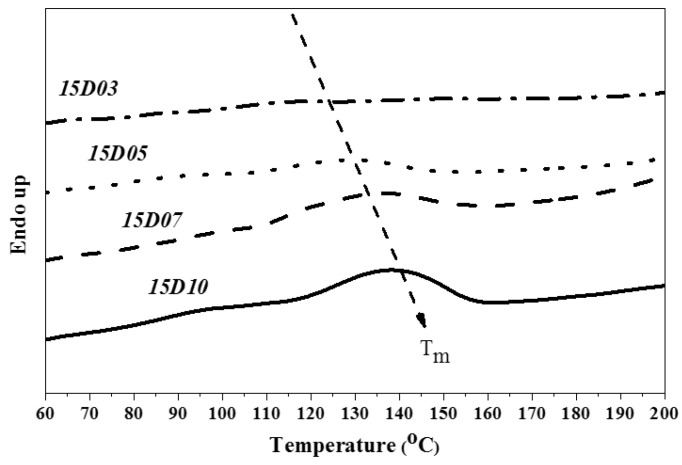
DSC curve of TPU with end-capping agent at concentration of 10 phm.

**Figure 15:**
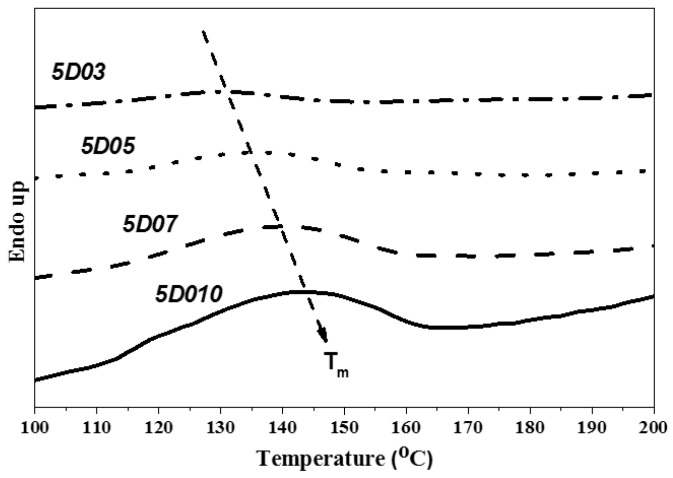
DSC curve of TPU with end-capping agent at concentration of 15 phm.

This study used PTMG and MDI as the base of synthesizing TPU, and used D03, D05, D07 and D10 as end-capping agents, respectively, to synthesize TPU. The hard segment melting point (Tmh) and melting enthalpy (ΔH) of TPU were analyzed by DSC. Figure 13, Figure 14 and Figure 15 show the DSC curve of synthetic TPU with three contents of end-capping agent. Table 5 showed the Tmh and ΔH data of TPU. The glass transition temperature (Tgh) of hard segment is not shown in the DSC curves. Because there are a few hard segments in relation to molecular chain length, the hard segment vitrification point is not obvious on the curve. As shown in Table 6, the DMAA concentration in the molecular structure of end-capping agent increased, and the Tmh and ΔH of TPU was also increased. The results are described below: (a) when the content of end-capping agent is 5.0 phm, the DMAA concentration in the end-capping agent increased from 3.0 wt% to 10 wt%, the Tmh of TPU rises from 131 °C to 144 °C, and ΔH increased from 0.8 J/g to 4.5 J/g; (b) when the content of end-capping agent is 10 phm, the DMAA concentration in the end-capping agent increased from 3.0 wt% to 10 wt%, the Tmh of TPU rises from 121 °C to 140 °C, and ΔH increased from 0.8 J/g to 2.6 J/g; (c) when the content of end-capping agent is 15 phm, the DMAA concentration in the end-capping agent is 3.0 wt%, and the Tmh is not obvious. When the DMAA concentration in the end-capping agent increased from 5.0 wt% to 10 wt%, the Tmh of TPU rises from 133 °C to 137 °C, and ΔH increased from 0.9 J/g to 2.2 J/g. The increase in Tmh resulted from the enhanced hydrogen bonding force between hard segments. The ΔH denoted the energy for complete phase transition of molecule segment of polymer. The molecular weight of polymer is proportional to the surface area of molecule, so the acting force increases with intermolecular contact area. The phase transition of molecular chain required higher energy. Therefore, the ΔH increased with the increasing of molecular weight of TPU. In the case of the same hard segment content, when the content of end-capping agent increased, the melting point of TPU shifted to low temperature, because the physical cross-linking points (H-bonds) of the molecular chain decreased with the molecular weight of TPU [29]; the trend is described below: (a) TPU synthesized with end-capping agent D03, the content of end-capping agent increased from 5.0 phm to 10 phm, the Tmh of TPU dropped from 131 °C to 121 °C, and the ΔH remains at 0.8 J/g; however, when the content of end-capping agent is 15 phm, there is no obvious Tmh endothermic peak in the DSC curve of TPU; (b) TPU synthesized with end-capping agent D05, the content of end-capping agent increased from 5.0 phm to 15 phm, the Tmh of TPU drops from 142 °C to 133 °C, and ΔH decreased from 1.3 J/g to 0.9 J/g; (c) TPU synthesized with end-capping agent D07, the content of end-capping agent increased from 5.0 phm to 15 phm, the Tmh of TPU drops from 140 °C to 137 °C, and ΔH decreased from 2.4 J/g to 1.8 J/g; (d) TPU synthesized with end-capping agent D10 series, the content of end-capping agent increased from 5.0 phm to 15 phm, the Tmh of TPU drops from 144 °C to 137 °C, and ΔH decreased from 4.5 J/g to 2.2 J/g.

### 3.5. Analysis of Glass Transition Temperature and Storage Modulus of TPU

**Figure 16:**
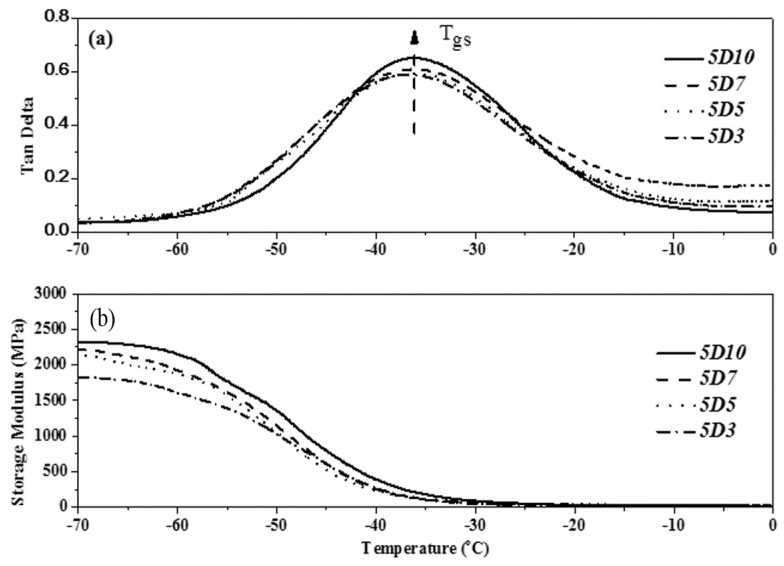
DMA curve of TPU with end-capping agent at concentration of 5.0 phm, (Effect of temperature on (**a**) Tan Delta (**b**) the storage modulus of TPU).

**Figure 17:**
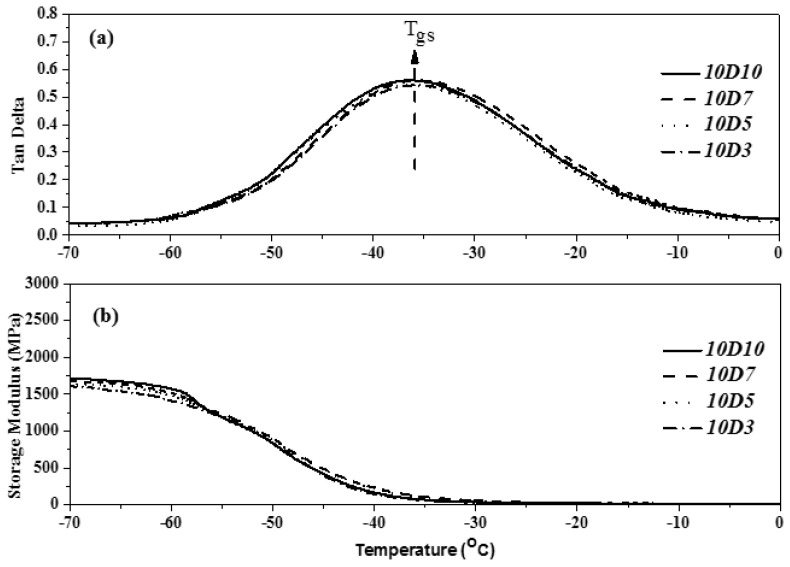
DMA curve of TPU with end-capping agent at concentration of 10 phm, (Effect of temperature on (**a**) Tan Delta (**b**) the storage modulus of TPU).

**Figure 18:**
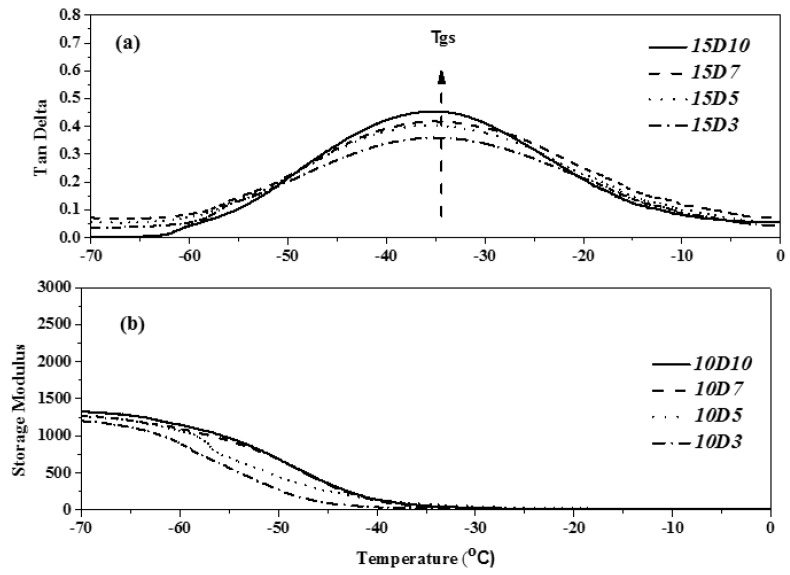
DMA curve of TPU with end-capping agent at concentration of 15 phm, (Effect of temperature on (**a**) Tan Delta (**b**) the storage modulus of TPU).

The DMA demonstrated the glass transition of various components of block polymer, provided the dynamic modulus of polymer and the law of variation of loss factor with temperature, and reflected the degree of microphase separation of the internal structure of polymer. The loss factor is the most effective method to judge the glass transition temperature of materials. The glass transition temperature of TPU generally referred to the glass transition temperature of soft segment (Tgs) [30,31]. This study used PTMG and MDI as the base of synthesizing TPU, and used D03, D05, D07 and D10 as end-capping agents, respectively, to synthesize TPU. The dependency relationship of the loss factor (tanδ) and storage modulus of TPU to the temperature were analyzed by DMA. Figure 16, Figure 17 and Figure 18 show the DMA curves of synthetic TPU with three contents of end-capping agent. According to the tanδ curves in Figure 16a, Figure 17a and Figure 18a, all of the TPU only showed one Tgs, meaning the PTMG, MDI and end-capping agent are mixed and continuous [32]. The results are described below: (a) when the content of end-capping agent is 5.0 phm, the DMAA concentration in the end-capping agent increased from 3.0 wt% to 10 wt%, and the Tanδ strength of TPU increased from 0.59 to 0.65; (b) when the content of end-capping agent is 10 phm, the DMAA concentration in the end-capping agent increased from 3.0 wt% to 10 wt%, and the Tanδ strength of TPU increases from 0.53 to 0.56; (c) when the content of end-capping agent is 15 phm, the DMAA concentration in the end-capping agent increased from 3.0 wt% to 10 wt%, and the Tanδ strength of TPU increased from 0.36 to 0.45. Because in the multiple H-bond states of hard segment, different acceptor and donor arrangements increase the H-bond strength of hard segment, the motion of soft segment is restricted [33,34]. As shown in Figure 14b, Figure 15b and Figure 16b, the storage modulus of TPU increased as the temperature dropped. The initial temperature is relatively high, the storage modulus of TPU approaches to zero, and the polymer chain has relatively strong motor ability; as the temperature drops, the resistance to segment motion increases, and the motor ability is weakened. When the temperature lowers to a certain level, the hard segments with relatively strong intermolecular force are gathered together, arranged in order and stacked closely. The crystals are formed, and the storage modulus increased continuously. However, the storage modulus of TPU changes as the DMAA concentration increased. The results are described below: (a) when the content of end-capping agent is 5.0 phm, the DMAA concentration in the end-capping agent increased from 3.0 wt% to 10 wt%, and the storage modulus of TPU increased from 1607 MPa to 2148 MPa; (b) when the content of end-capping agent is 10 phm, the DMAA concentration in the end-capping agent increased from 3.0 wt% to 10 wt%, and the storage modulus of TPU increases from 1416 MPa to 1517 MPa; (c) when the content of end-capping agent is 15 phm, the DMAA concentration in the end-capping agent increased from 3.0 wt% to 10 wt%, and the storage modulus of TPU increased from 904 MPa to 1148 MPa. Because the molecular chain motion of soft segment of TPU is restricted by hard segment, the rigidity of TPU is increased, and then the storage modulus is increased [35,36].

## 4. Conclusions

Based on the above discussion, this study used the end-capping agent to reduce the molecular weight of TPU and to adjust the DMAA concentration in the end-capping agent, which ultimately promoted the degree of H-bonding of TPU. Moreover, the contradictory problem in molecular weight control, mechanical properties and coating temperature of TPU is solved successfully. The findings showed that the FT-IR spectra verified the TPU having end-capping agent, PTMG and MDI, which completely reacted with each other. The DMAA in the end-capping agent effectively promoted the hydrogen bonding degree of TPU. However, the steric hindrance effect of DMAA influenced the chain termination reaction, so that the molecular weight of TPU is changed. In terms of thermal property, the DMAA promoted the molecule segment of TPU to synthesize the complex hydrogen bonding network. It was observed that when pyrolysis temperature of TPU is increased, the melting point and ΔH of the hard segment are also increased. Due to the polarity difference between the hard segment and soft segment with end-capping agent, the hard segment is difficult to be mixed in the soft segment phase, and the Tgs temperature remains stationary. When the temperature is low, the hard segments with relatively strong intermolecular force are gathered together, arranged in order and stacked closely. The crystals are formed, and the storage modulus increased continuously. The AFM verifies that the micro phase separation degree increases with the end-capping agent content, and irregularly shaped hard domains are generated and connected to each other. Finally, the hydrogen-bond acceptor (DMAA) concentration in the molecular structure of end-capping agents was adjusted in this study, which is an effective strategy for good mechanical properties of low molecular weight polymers. The mechanical properties of seamless bonding with nylon substrate will be analyzed and various application sides will be tested in subsequent research.

## Figures and Tables

**Table 1:** Formula for synthesis of end-capping agent.

Code	End Capping Agent Synthesis Monomer					Molecular Weight Analysis of End Capping Agent		
	MMA (wt%)	DMAA (wt%)	EAC (wt%)	AIBN (phm)	ME (phm)	Mw (g/mole)	Mn (g/mole)	PDI (Mw/Mn)
D03	37	3	60	0.4	30	1070	943	1.13
D05	35	5	60	0.4	30	1032	928	1.10
D07	33	7	60	0.4	30	1016	906	1.13
D10	30	10	60	0.4	30	1013	896	1.12

Phm is a unit symbol for the concentration of a liquid, which is 1% ppm.

**Table 2:** Formula for synthetic TPU.

Sample Code	End-Capping Agent Model	PTMG (wt%)	MDI (wt%)	End-Capping Agent (phm)
5D03	D03	75	25	5
5D05	D05	75	25	5
5D07	D07	75	25	5
5D10	D10	75	25	5
10D03	D03	75	25	10
10D05	D05	75	25	10
10D07	D07	75	25	10
10D10	D10	75	25	10
15D03	D03	75	25	15
15D05	D05	75	25	15
15D07	D07	75	25	15
15D10	D10	75	25	15

**Table 3:** Area percent data of simulated peak splitting of –C=O and –NH.

Sample	Carbonyl Region				Amine Region			
	–C=O _(H-bond)_		–C=O _(free)_		–NH _(H-bond)_		–NH _(free)_	
	Wave Number (cm^−1^)	Peak Area (%)	Wave Number (cm^−1^)	Peak Area (%)	Wave Number (cm^−1^)	Peak Area (%)	Wave Number (cm^−1^)	Peak Area (%)
10D3	1711	41.7	1732	58.3	3300	70.0	3440	30
10D5	1711	43.6	1732	56.4	3300	76.0	3440	24
10D7	1711	50.5	1732	49.5	3300	79.0	3440	21
10D10	1711	57.6	1732	42.4	3300	81.0	3440	19

**Table 4:** Effect on Mw and Mn of TPU by varying the concentration of DMAA in the end-capping agent and Effect on Mw and Mn of TPU by varying the concentration of end-capping agent.

End-Capping Agent (phm)	DMMA	Mw (g/mol)	Mn (g/mol)	DPI (Mw/Mn)
5	3.0 wt%	82,000	35,520	2.31
	10.0 wt%	111,580	56,100	1.98
10	3.0 wt%	63,460	28,270	2.24
	10.0 wt%	91,540	47,020	1.94
15	3.0 wt%	52,480	27,960	1.88
	10.0 wt%	68,080	37,940	1.80
**Code**	**End-Capping Agent** **(phm)**	**Mw** **(g/mol)**	**Mn** **(g/mol)**	**DPI** **(Mw/Mn)**
D03	5	82,000	35,520	2.31
	15	52,480	27,960	1.88
D05	5	86,810	41,530	2.10
	15	55,080	26,020	2.12
D07	5	96,330	47,980	2.00
	15	64,730	28,300	2.28
D10	5	111,580	56,100	1.98
	15	68,080	37,940	1.80

**Table 5:** Pyrolysis temperature property data of TPU.

Sample ID	5D03	5D05	5D07	5D10
T_d5_ (°C)	312	328	322	332
T_d10_ (°C)	330	343	336	346
decomposition rate (%/°C)	0.80	0.83	0.83	0.86
Sample ID	10D03	10D05	10D07	10D10
T_d5_ (°C)	308	312	320	325
T_d10_ (°C)	325	329	337	341
decomposition rate (%/°C)	0.76	0.70	0.80	0.90
Sample ID	15D03	15D05	15D07	15D10
T_d5_ (°C)	309	317	320	323
T_d10_ (°C)	324	335	337	342
decomposition rate (%/°C)	0.71	0.83	0.83	0.90

**Table 6:** DSC data of TPU.

Code	5D03	5D05	5D07	5D10
T_mh_ (°C)	131	142	140	144
ΔH (J/g)	0.8	1.3	2.4	4.5
Code	10D03	10D05	10D07	10D10
T_mh_ (°C)	121	131	136	140
ΔH (J/g)	0.8	1.2	2.1	2.6
Code	15D03	15D05	15D07	15D10
T_mh_ (°C)	--	133	137	137
ΔH (J/g)	--	0.9	1.8	2.2

## Data Availability

Not applicable.
